# The effect of catchment based mentorship on quality of maternal and newborn care in primary health care facilities in Tigray Region, Northern Ethiopia: A controlled quasi-experimental study

**DOI:** 10.1371/journal.pone.0277207

**Published:** 2022-11-17

**Authors:** Haftom Gebrehiwot Weldearegay, Alemayehu Bayray Kahsay, Hagos Godefay, Pammla Petrucka, Araya Abrha Medhanyie

**Affiliations:** 1 Mekelle University, College of Health Sciences, Mekelle, Ethiopia; 2 Tigray Region Health Bureau, Tigray, Ethiopia; 3 University of Saskatchewan, College of Nursing, Saskatoon, Canada; 4 Nelson Mandela African Institute of Science and Technology, Arusha, Tanzania; Jhpiego, UNITED STATES

## Abstract

**Background:**

In Ethiopia, quality of maternal and newborn care is poor. This situation has persisted, despite the wide implementation of several capacity building-oriented interventions including clinical mentoring for skilled birth attendants that were anticipated to translate in to high–quality maternal and newborn care on each encounter. The effectiveness of mentoring programs is not yet well documented in the research literature. Therefore, we evaluated the effect of a catchment based clinical mentorship in improving the quality of maternal and newborn care in primary level facilities of Tigray, Northern Ethiopia.

**Methods:**

We conducted a controlled quasi-experimental pre-post study among 19 primary health care facilities, with 10 facilities assigned to the group where the catchment based clinical mentorship program was implemented (intervention group), and 9 facilities to the control group. We assigned the group based on administrative criteria, number of deliveries in each facility, accessibility, and ease of implementation of the intervention. A sample of 1320 women(662 at baseline; 658 at post intervention) and 233 skilled birth attendants(121 at baseline and 112 at end line) were included. We collected data from mothers, skilled birth attendants and facilities. The first round of data collection (baseline) took place two weeks prior the inauguration of the intervention, 05 October to 04 November 2019. The end line data collection occurred from 22 May to 03 July 2020. The primary Outcome was “receipt quality of maternal/newborn care”. We analyzed the data using difference in differences (DiD) and logistic regression with Generalized Estimating Equation. The level of significance of predictors was declared at p-value less than 0.05in the multivariable analysis.

**Intervention:**

We deployed a team of local clinical mentors working at primary hospitals to provide clinical mentorship, and direct feedback in routine and emergency obstetrical and newborn care to the mentees (all skilled birth attendants performing maternal and newborn health services) functioning in their catchment rural health centers for duration of six months. While visiting a facility, mentors remain at the facility each lasting at least five to seven days per month, over the course of intervention period.

**Results:**

A significantly higher proportion of women at intervention facilities received quality of care services, compared with women at comparison facilities. (DiD = 18.4%, p<0.001). Moreover, following the implementation of the intervention we detected a difference in the occurrences of maternal complication outcome during delivery and immediately after birth. This was decreased by 4.5%, with significant differences between intervention and comparison sites (DiD = 4.5%, p = 0.013). We also found a favorable difference in occurrences of neonatal obstetric complications, with a decrease of 4.8% in the intervention site and almost no change in the comparison site (DiD = 4.8%, p = 0.002). Among the determinants of quality of care, we found that providers’ job satisfaction (AoR = 2.95, 95%CI: 1.26 to 6.91), and making case presentation at regular basis(AoR = 1.89, 95%CI: 1.05 to 3.39) were significantly associated to improve the quality of care. However, delivery load(AoR = 0.95, 95%CI: 0.93 to 0.98) was negatively associated with quality of care.

**Conclusions:**

We conclude that the catchment based clinical mentorship intervention is effective to improve quality of care and reduce childbirth complications in northern Ethiopia. This finding further elaborated that incorporating maternal and newborn health catchment based clinical mentorship activities into the existing health system strengthening strategies can catalyze improvement processes to quality practice and health systems. This is seen as a necessary step to achieve the effective quality universal health care required to meet the health-related Sustainable Development Goals. Besides, more attention needs to be given to develop interventions and strategies that directly enhance providers’ job satisfaction and reduce delivery work load.

## Introduction

Improving the quality of maternal and newborn care remains a priority in today’s world [1, 2]. Inadequate health-care provider performance is also a major challenge to the delivery of high-quality health care in low-income and middle-income countries [3]. Specifically, Ethiopia has achieved remarkable success in reducing maternal and neonatal mortality in recent decades, but still has high neonatal mortality rate (29 deaths per 1,000 live births) and maternal mortality ratio (412 deaths per 100,000 live births). This progress has been partly due to a rise in institutional births influenced by the commitment of Government of Ethiopia’s flagship programs [4, 5]. However, recent evidence suggests that the available maternal and newborn health services are of poor quality [4–7]. A significant proportion of maternal and newborn mortalities sadly occur during delivery and the immediate postpartum period in health facilities that cannot always guarantee care that is ‘effective, safe, client-centered, delivering services that are timely, equitable, integrated, and efficient’ as recommended by the World Health Organization (WHO) [2, 8]. Moreover, with the ambitious Sustainable Development Goals to reach by 2030, it becomes clear that efforts should now be directed towards better quality of care (QoC), in addition to the further expansion of health services coverage [9, 10]. This calls for attention to the QoC in order to address avoidable maternal and newborn morbidity and mortality [11, 12].

Nevertheless efforts within Ethiopia to implement quality improvement programs in the past have met with limited success. While government recognize the need to equip facilities with skilled staff and provide resources to improve the QoC, factors persist that reduce the overall quality of maternal and newborn care, such as inadequate, and inappropriate pre-service training, often limited by gaps in individual skilled birth attendants’ competencies’ and provision of poor continuity of care [7, 13]. Poor caring behaviors, inability to translate training into practice and poor providers’ level of adherence to good practices and standards, especially at the primary health facilities is well documented in Ethiopia [7]. Furthermore, in Ethiopia, in-service training is widely instituted to upgrade the knowledge and clinical skills of skilled birth attendants (SBAs)that can then translated in to high–quality care for both the mother and newborn at each encounter. But, merely training more SBAs will not suffice: rather more attention needs to be directed at their behaviors, clinical skills, and how these translate to the care that SBA provides for women and newborns [9, 14, 15]. Therefore, targeted, well programmed catchment based clinical mentorship intervention is preferable to one-time training and leads to the sustained use of new skills which in turn to yield sustainable high-quality clinical care outcomes. Catchment based clinical mentorship (CBCM)is a system of practical training and consultation that enables health care provides to practice new skills in clinical settings with the support and guidance of a more specialized and experienced clinician to yield sustainable, high-quality clinical care outcomes [16]. CBCM is a newly strategies where both mentors and mentees work in health facilities that have direct referral linkage within a catchment [7, 17, 18]. Besides, CBCM of SBAs could be one of the best strategies that have not been tried in the public health system in Ethiopia on large scale but field reports shown that as beneficial for professional growth and competency development amongst novices in the health field, with presumable robust gains to the whole health care delivery system [7, 17, 19]. However, the effectiveness of CBCM intervention to improve QoC of mothers and newborns in primary health care settings is not yet well documented in the scientific literature. To address this paucity of evidence, we designed and tested a catchment based clinical mentorship intervention among SBAs to improve the QoCat primary health centers (PHC) of Tigray, Northern Ethiopia, since PHCs are the first point of contact for institutional births, and have received lot of attention and investment by the Ethiopian Federal government. One of the major attention and contribution to the gains in the health sector is the Health Extension Program, our flagship community-based primary health care (PHC) delivery platform; has proven to be an effective intervention by serving as the largest component of Ethiopia’s health care delivery system in terms of reach and thus transforming access to health care services. Consider the situational context of rural setting of Tigray, northern Ethiopia, this research team conceptualized a program to train a group of local clinical SBA (professionally qualified providers with midwifery skills* (midwife, health officer, nurse or doctor) who has been trained to proficiency in the skills necessary to manage normal deliveries and diagnose, manage, or refer obstetric complications) to mentor other SBA clinicians working with in their catchment area health facilities [20–22]. Consequently, this study could generate and adds new evidence to the body of literature and inform health care policy-makers about the effectiveness of the intervention on QoC and will serve as a starting point to improve the quality gap in maternal and newborns services in Ethiopia.

## Materials and methods

### Study design and setting

We conducted a quasi-experimental study design using controlled before-and-after study among 19 primary health care facilities (four primary hospitals as a center for mentors and 15 Health Centers) in South east zone of Tigray regional state, Northern Ethiopia. All rural health centers within the study setting were included. Consequently, ten facilities were assigned to the catchment based clinical mentorship implementation (i.e., intervention group), and nine facilities were assigned to the group without catchment based clinical mentorship implementation (i.e., control group).

Selection and assignment of the health facilities in to groups was not performed randomly but based on the administrative criteria, ability to reach facilities with ease, number of deliveries in each facility, and ease of implementation of intervention (i.e, selecting health facilities that are near one another to ease mentor travel or within their catchment site). Primary hospitals were evenly split between control & intervention groups; as there is one primary hospital exist per district. District/Woreda health offices oversee and coordinate primary care services for catchment areas of approximately200,000 populations, including oversight of five to six health centers, 20–30 health extension workers and in most cases, a primary hospital. Ethiopia’s health service is structured into a three-tier system: primary, secondary and tertiary levels of care. The primary level of care includes primary hospitals, health centers (HCs) and health posts (HPs). The primary health care unit (PHCU) comprises five satellite HPs (the lowest-level health system facility, at village level) and a referral HC. This is the point where the primary health centers are administered and primary services facilitated under the health service delivery structure [23].

### Sample size calculation and sampling technique

Before the implementation of the intervention, a facility based survey was conducted to assess the level of quality of maternity care in Tigray, Northern Ethiopia. The sample size was determined using a two-sided Z test of the difference between proportions with 80% statistical power, n2/n1 (ratio between the intervention and control areas 1:1) a 5% significance level. The outcome of interest used in the calculation of the sample size was the proportion of mothers who received quality of maternity care which was 20.7% from the baseline survey in northern Ethiopia [21]. By assuming the effect size to be increased by a 10% increases in the number of mothers and newborns who report receiving high-quality care after a half year implementation of the intervention with unequal cluster size evaluation. The design effect was 1.5 which was calculated by considering the intra-class correlation (roh) = 0.05 and coefficient of variation (CV) = 0.10 [24, 25]. Based on the above parameter assumption considering a 10% upward adjustment for possible non-response, 662 mothers who delivered at the facility were sampled for the baseline survey. A total of 1320 mothers were included both for the intervention and comparison groups in the baseline and end line surveys.

**Fig 1:**
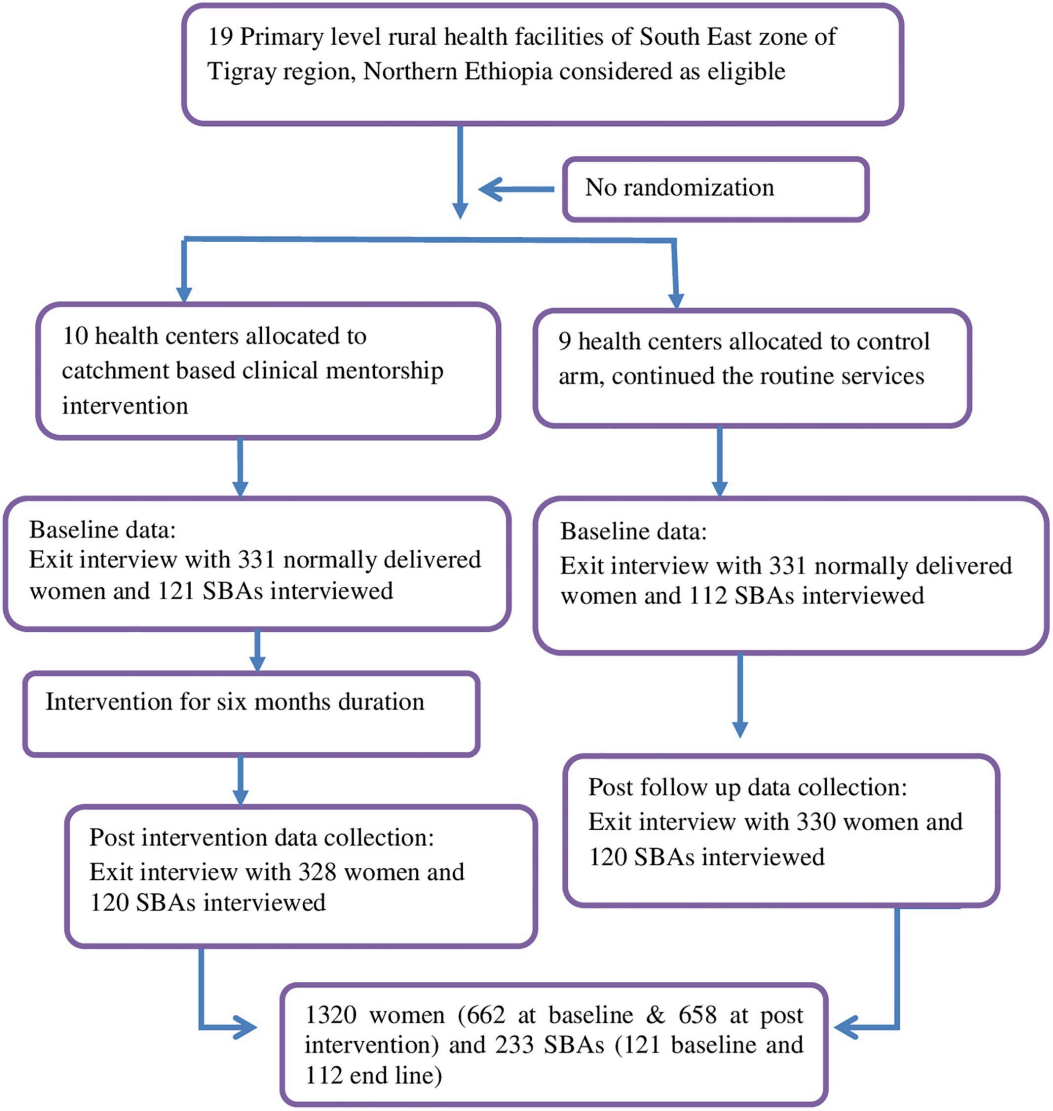
Pre-post quasi experimental with comparison group study design sampling protocol.

Regarding the sampling technique, the South east zone of Tigray Regional State which had four districts (known as woredas) selected. Woredas are the third level administrative division of the country, following regions and zones. There is one primary hospital exist per district. Thus, two districts (i.e., Hagereselam and Hintalo-Wajerat) were assigned to the intervention project site, and the other two districts (i.e., Enderta and Samre) were assigned to the group without implementation (i.e., control site). With regards to study participants’, only mothers experiencing uncomplicated vaginal deliveries admitted before or on the active stage of labor phase and who received basic emergency obstetrics care were interviewed at time of exit. This was conduct in the form of user exit surveys at the point of service delivery. All SBAs in the study were enrolled. Finally, all eligible recently delivered women were chosen by a systematic random method until the required sample size was achieved. Laboring women who required Caesarean birth were excluded from the study as well as referral case mothers. The sampling protocol of this controlled quasi-experimental study design is reflected in the flow diagram “Fig 1”.

### Development of Catchment Based Mentorship (CBCM) intervention test model and underlying assumptions

Our theory of change or test model of the quality improvement intervention postulated that catchment based clinical mentorship would contribute to improved SBAs performance and maternity care quality, ultimately leading to better maternal and newborn health outcomes. This CBCM program developed from a combination of the WHO model of QoC [16] and UNICEF’s Every Mother Every Newborn guide for quality improvement [26] and Ethiopia Federal Ministry of Health’s Catchment-based Reproductive, Maternal, Newborn, child, and Adolescent health (RMNCAH) national guideline [27]. We have adapted with some modification in conjunction with Tigray Regional Health Bureau experts and provincial stakeholders as the intervention is a newly approach in Ethiopia and a basic foundation for quality improvement in maternity care services.

The interventions’ mandate was threefold: (1) to continuously optimize competency and performance of SBAs given the resources at hand, the context of Ethiopia. (2) to generate evidence of effectiveness of CBCM on quality of maternal and newborn care, and (3) to reveal the process of scaling up a mentoring program across the region. This mentorship intervention was designed to cover the basic MNH or obstetrics signal functions [9].

Besides, research team members and Regional Health Bureau experts jointly selected SBA clinical mentors and regional level supervisors under the umbrella of the regional Maternal Newborn Child and Adolescent Health Task Team. A mentor (an experienced person) allows a mentee (a less experienced person) to gain and develop knowledge, abilities, and maturity in a specific position or a professional area that they share [27].

The local mentors recruited from Hagereselam and Hintalo-Wajerat intervention site districts in South East zone of Tigray Regional State. Recruitment of SBA mentors was done through open advertisements. The detailed recruitment and selection criterion for local SBA mentorship is found in the S1 Appendix. Additionally, five days training on the CBCM intervention and quality improvement mechanism was provided.

Furthermore, the supervisors and local mentors met monthly on a regular basis, and a regional work shop was organized with experts, mentors, and mentees in clinical mentoring to oversee, monitor, and implement the program. The supervisors’ role was to coordinate and supervise the mentors’ field activities during their routine coaching of the SBAs and health facilities during the clinical mentorship implementation period.

### Implementation of the intervention

The CBCM intervention or program is a new approach to clinical mentorship. The CBCM is a process providing practical training to clinical staff in order to enhance the competency of providers which in turn potentially improves QoC services in LMIC contexts [16]. This intervention was the provision of catchment based, group on-site mentoring offered at the individual mentee`s duty station through periodic on-site visits. On-site mentoring is recommended since it allows mentees to learn while continuing to provide services in their respective duty stations. However, if on-site mentoring is found to be difficult, it is possible to hybridized it with an off-site approach or even off-site mentoring alone (i.e., placing mentee at mentor`s facility) [28, 29]. It is important to note that the catchment is set up based on the health center’s geographical proximity to the mentoring health facility. In this thesis, primary hospitals were considered to have the primary responsibility to mentor the health centers within their catchment.

Mentoring was begun after the baseline data collection was completed. Consequently, we hired and trained a team of six qualified local SBA mentors, who work in health facilities that have direct referral linkage within a district or catchment to facilitate the mentorship sessions in the intervention site after they receive a refresher CBCM training to other SBA staff at PHC. After that, at the intervention facilities, one mentor was responsible for visiting one or two facilities over the course of the six month clinical mentoring period. While visiting a facility, mentors remain at the facility for the duration of each clinical mentoring session. Mentors conducted a minimum of six mentoring sessions, each lasting at least five to seven days per month over the course of intervention period, for each facility. When possible, they stay overnight at facilities which optimizes mentoring time and by minimizing travel time to remote facilities, thereby strengthening relationships with facility staff. Clinical mentors conduct side-by-side observation and mentoring on management of clinical cases. During their visits, mentors provide immediate feedback on individual and systems performance and review overall findings and recommendations with mentees and the health center (HC) director.

In general at each facility, the clinical mentors were responsible for: (1) strengthening mentees’ knowledge, attitudes, clinical skills and clinical decision-making; (2) assessing the routine evidence based care practices mainly the core clinical competencies of basic emergency obstetric and neonatal care; (3) guiding mentees through a re-examination of their ideas and values; and (4) ensuring the learning and personal or professional development of mentees. (5) Providing effective feedback to mentees: and (6) determine if performance standards are being met that improve QoC of mothers and newborns. In addition to in-person mentoring, mentors were accessible by phone to provide advice or answer mentees’ patient care questions at any time during the mentoring time [30].

### Monitoring and evaluation of implementation of the intervention

Before the start of this CBCM intervention, selected mentors were trained in quality assurance and in clinical mentorship by the experienced local mentors, who are regional experts in Northern Ethiopia. During the implementation period, the performance of the clinical mentors and mentees was monitored and evaluated. The key monitoring questions were: (1) Is the implementation of the catchment based clinical mentorship program being conducted as planned? (2) Are the clinical mentors performing according to the standards? (3) Are the mentees performing according to the expectations? The supervisors were responsible for sending a monthly summary report of clinical mentors’ visits to the facilities, including challenges, lessons, and best experiences of the clinical mentoring sessions, activities, and qualitative issues noted by the clinical mentor.

**Fig 2:**
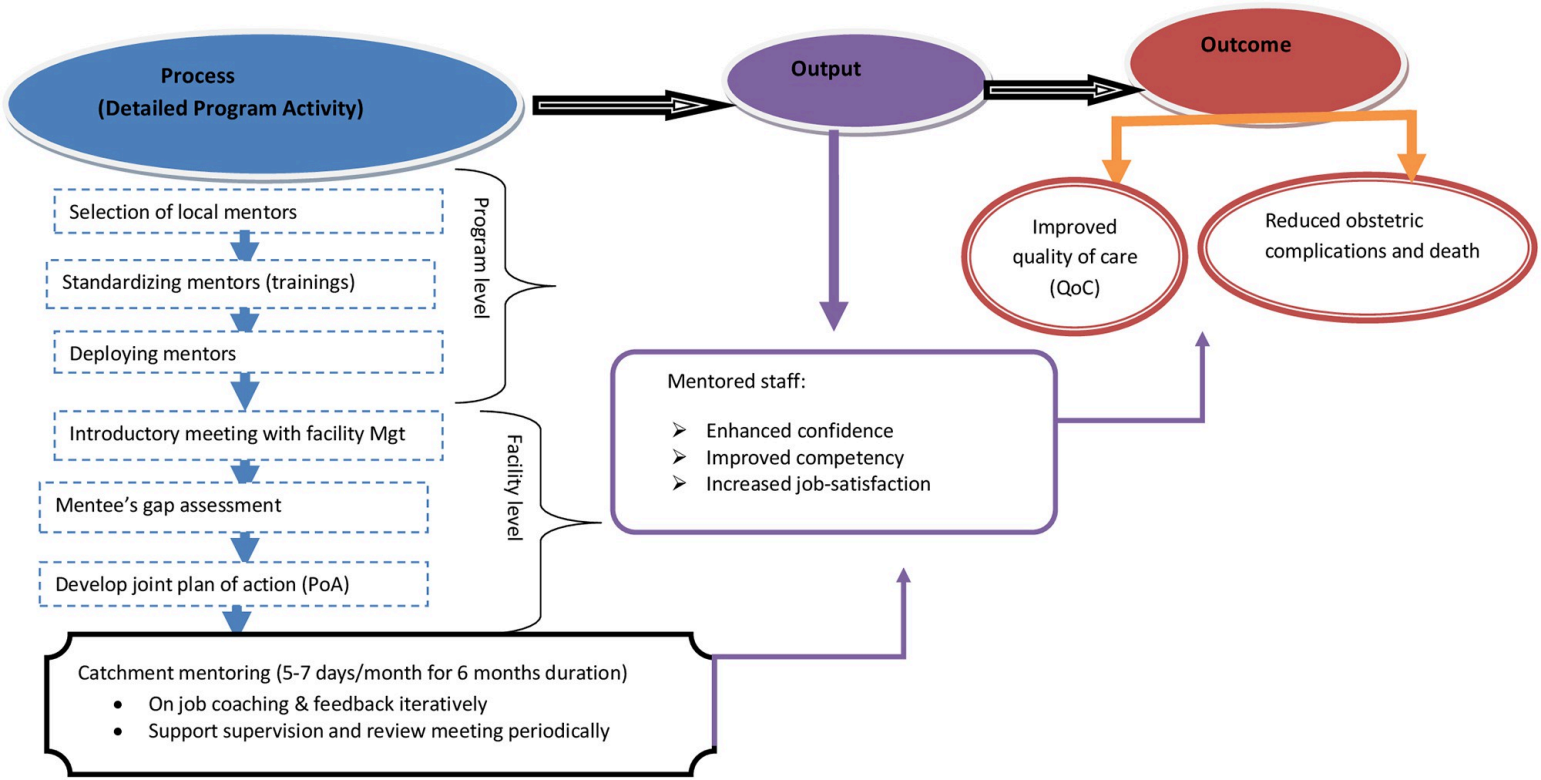
Theory of change for catchment based clinical mentorship intervention to improve QoC in primary level facilities of Tigray region, Ethiopia.

Moreover, assessment of the mentorship program was done by conducting periodic supportive supervision as well as review meetings. Review meetings were conducted every 3 months at Regional Health Bureaus level and every two months at the district/catchment level to share their best experiences and evaluate progress of the programs. Finally, at the end of the program we evaluated the overall effect of a catchment based clinical mentorship in improving the quality of maternal and newborn care in primary level facilities. Theory of change for catchment based clinical mentorship intervention is found in “Fig 2”.

### Study participants and data collection

The sources of data for this study were clients, skilled birth attendants, and facilities **[Table 1]**. The data collection instruments or questionnaires were translated into the appropriate local language “Tigrigna” and underwent minor modifications to improve local understanding and clarity for participants. Moreover, for few of the technical questions special emphasis was given in how the mothers could be easily understood by their local language expressions.

**Table 1:** The source of data with along its description on the method of data collection for baseline and end line survey in Tigray region, Northern Ethiopia.

Source of data	Description of means of data collection
**Clients’**	The sample from clients’ refers recently delivered women in the health facility.
	The data were collected via face–to—face interviews. This was conducted in the form of user exit surveys at the point of service delivery.
**Skilled birth attendants’**	SBAs were eligible if they were working in MNH services of the study facilities and fully recruited staff, excluding students on apprenticeship. If SBA were unavailable on the day the study team visited, the study team arranged to come at a later date to give the providers an opportunity to participate in the survey.
	The basic emergency and obstetric knowledge test, job satisfaction and clinical vignettes related questions were self-administered questions adapted from a national basic emergency obstetrics and newborn care training manual [32]. SBAs were instructed to respond to the scenario as if they had access to all of the resources they needed and were asked questions about what they would ask or do for a hypothetical patient.
**Health facilities’**	The facility inventory which focused on: basic facility information, such as facility identification and cadre of employees; and infrastructure; availability of equipment, supplies, and medicines.
	The most senior healthcare provider available on the day of interview, usually the ‘facility-in-charge’, was asked to respond to the survey. Research assistants verified the availability of equipment, supplies, and medications; the respondent confirmed stock outs.

In general, baseline data collection took place two weeks prior to initiating the intervention, 05 October to 04 November 2019. The end line data collection occurred from 22 May to 03 July 2020. The data were collected by fourteen research assistants who were health care providers with working experience in maternal and newborn health (MNH) care, On-site supervision provided by a research supervisor. The data collectors received extensive preparatory training on the study objectives, data collection procedure and skill, informed consent, as well as on the principles of catchment-based mentorship approach intervention and evidence based essential obstetric practices. To ensure that interviews were done in a similar manner, a validated quality indicator tool was used. Details of the measurement and validated tool findings are found in a previous paper published elsewhere [31].

### Variables and measurements

The primary outcome investigated was receipt of QoC. It was measured as a continuous variable constructed as a composite variable from the total of 32 standards of quality process of care indicators. The QoC signal functions used in this study are grounded in validated indicators in the Tigray regional state context, found in a previous paper published elsewhere [31]. Secondary outcome were maternal and neonatal complications. Therefore, quality measures reflect the minimum standards of routine intra-partum and immediate postpartum care, in relation to the group of health facilities (intervention and comparator) where the delivery service is performed. Accordingly, QoC was defined as a binary variable of “adequate” versus “in adequate”. If a mother received 75% and above, it was termed as received adequate QoC, otherwise inadequate QoC was assessed. Maternal and neonatal outcomes were secondary outcomes of interest which were measured as any occurrences of maternal and newborn complications either during or immediately following delivery, including:bleeding,preeclampsia/eclampsia, laceration, birth asphyxia, still birth, infection, maternal or and newborn death within the health facility and another type of complication (asked to specify).

The providers’ satisfaction variable was classified as “satisfied” (providers scored 75th percentile and above), whereas below the 75th percentile was considered “not satisfied”. Facility readiness was categorized as adequately ready at the 75th percentile and above and below was considered inadequately ready). Knowledge were evaluated out of 100, and grouped as either had adequate knowledge (median or higher) or inadequate knowledge (less than median value). Clinical vignette competency of SAs was evaluated out of 100, and grouped as either competent (median or higher) or not yet competent (less than median value).

The independent variables were group status (intervention/ control), demographic characteristics, SBAs” knowledge, clinical vignette, job satisfaction, MNH related training, cadre, fear of legal issue to make decision, making regular case presentations and promoting regular staff rotation and facility characteristics. In addition, the covariate variable of delivery load on a weekly basis was also included in the regression model.

### Statistical analyses

Data was entered in to EpiData^™^ 3.1 software. Balance intergroup difference or homogeneity test to confirm the identical nature of the SBAs data were done before the intervention. S2 Appendix shows the results of the chi-squared test of homogeneity between intervention and comparison groups from the figures of SBA sat baseline survey. In this situation, there was no statistically significant difference of the principal predictors among the groups, allowing for an unbiased estimate of treatment effect. This alternative approach of matching controlled the delimitation of selection bias and confounding and allowed the research to proceed without conducting a propensity score-matched analysis to get a valid net effect of the intervention. Then descriptive analysis was undertaken to describe the study population in relation to socio-demographics, each of the quality indicators and other relevant variables.

We conducted difference-in-differences (DiD) analyses assessing the difference between intervention and control facilities in the change of population average quality of care from baseline to end line. These analyses control for both differences in quality patterns between facilities at baseline and changing patterns over time those are external to the intervention, but consistent across the facilities, fulfill the criteria of parallel trend assumption. To obtain an appropriate estimate of the effect of a specific intervention or treatment by comparing the changes in outcomes over time between a population that is enrolled in a program (intervention group) and a population that is not (control group). We included a fixed effect for facilities to account for the correlations between subject responses during the design phase. In this study, subjects within the cluster are more likely to interact, sometimes receiving care from a single health care provider which can lead to a loss of statistical power. Calculating intra-cluster dependence can be quantified by considering the intra-cluster correction coefficient (ICC). The facility cluster effect is mostly below the threshold (ICC less than 0.05) which is significant in our case, as it implies there is a cluster effect, which in turn signified the need for use of the Generalized Estimating Equation (GEE) analysis method. Furthermore, the nature of the outcome variable was a dichotomous outcome; binomial distribution and logit link were used to estimate the odds ratios, predicted probabilities, and 95% confidence intervals. We assumed the order of responses within a cluster was arbitrary or no logical ordering of observations. The model-based estimator was considered to account for clustering at the facility level and main effect was the term used to build the reported model. Therefore, logistic regression with the GEE analysis method with an exchangeable correlation structure with the lowest QIC score (closest to zero) was used. Bivariate analysis was used to determine the strengths of the association between the independent variables and the primary outcome variable. Multivariable logistic regression with GEE was used to identify factors independently associated with quality of care. The level of significance of predictors was declared at p-value less than 0.05 in the multivariable analysis.

### Ethics approval and consent

The research protocol was approved by the Institutional Review Board (IRB) of the College of Health Sciences at Mekelle University (Protocol number 1436/2018). Additionally, permission was secured from the Regional Health Bureau and relevant local government offices. Once potential participants decided to be included in the study, data collectors secured a written consent form and proceeded with the interview. Only individuals who have freely consented participated in the study, and no coercion or deception were used. The written consent form used for the study details the aims, methods, and anticipated benefits and risks of the research. It also informs the respondents that their identity and replies will be confidential, and that participating in the study will not result in any potential hazards or impacts on their health care service.

Lastly, the participants are also informed that they have the right to abstain from the study, or to withdraw at any point. Data collection was conducted confidentially and data were de-identified and de-linked, with data storage being in a secure location.

## Results

Overall, 662 women (331 in each of the intervention and control group), at baseline and 658 women (328 in the intervention and 330 in the control) at follow-up were included in this study.

The average age of the women was 28.2 (±SD 6.4) years, similar at baseline and end line in both intervention and control facilities. Among the intervention group nearly two thirds at baseline and 91.2% of the mothers at end line had birth preparedness and complication readiness plans. Both at baseline and end line of the comparator group more than nine out of ten mothers had ANC visits during for their last pregnancy. In both the intervention and control groups less than 10% at baseline, and at end line, more than one third of the mothers stayed in the facility for more than 24 hours after normal delivery. More than four out of five mothers across both times of the survey across the groups had no complications during their current labour and delivery. Among intervention group about 10.3% at baseline and 4.0% of the mothers at end line had neonatal complication during their current labour and delivery service. This study finding again revealed that, among the intervention within the group who had a neonatal complication, nearly 3% and 0.8% of them were still birth at baseline and post intervention respectively [Table 2].

**Table 2:** Descriptive statistics of mothers who delivered in the primary health care facilities, a baseline and end line survey across the intervention and control sites of Tigray region, Northern Ethiopia,2020 (N = 1320).

Demographics	Baseline(n = 662)		End line(n = 658)	
	Control(n = 331) (Mean, or percent)	Intervention(n = 331) Mean, or percent	Control(n = 330) Mean or percent	Intervention(n = 328) Mean or percent
Age(mean)	29.15	28.83	27.57	27.38
Education				
**No formal**	40.5	61.0	61.8	51.5
**Elementary**	32.9	25.1	22.7	29.6
**Secondary and above**	26.6	13.9	15.5	18.9
Occupation				
**Housewife**	72.2	68.0	84.2	84.8
**Employed**	20.8	23.3	11.8	11.3
**Daily worker**	6.9	8.8	3.9	4.0
Marital status				
**Married**	88.8	85.8	90.3	94.2
**Single/never married**	5.7	4.5	5.5	3.0
**Divorced/widowed/separated**	5.4	9.7	4.2	2.7
Walked time to the nearest health facility	1 hour	1 hour and 30 min	1 hour and 20 min	1 hour and 15 min
Mother had birth preparedness and complication readiness plan (BPCR)				
**Yes**	45.3	65.6	81.2	91.2
Mother had ANC visit for the last pregnancy				
**Yes**	87.9	93.4	95.2	96.6
Length of labor and delivery in hours (mean)	8.0	7.0	6.2	7.0
Mothers stayed in the facility for more than 24 hours following normal delivery	8.2	6.9	35.8	34.8
Number of gravidity (mean)	2.95	3.02	3.0	3.1
Number of parity (mean)	2.76	2.84	2.97	3.19
Mother had a history of stillbirth, at some point in their lives				
**Yes**	12.1	10.0	7.6	8.8
Partner being allowed to enter to the delivery room				
**Yes**	53.2	40.5	32.1	55.2
Mothers had involved in their care decision				
**Yes**	71.9	76.7	77.9	89.9
Mothers had complicated during their current labour				
**Yes**	11.5	13.9	13.6	11.3
Type of maternal obstetrics complication(s)				
**Hemorrhage**	3.9	2.1	6.7	6.1
**Pregnancy induced hypertension**	4.5	4.8	3.6	2.7
**Infection**	1.0	6.0	2.7	1.5
Other complications*	2.1	1.0	0.6	1.0
Neonatal complication				
**Yes**	10.6	10.3	3.9	4.0
**No**	89.4	89.7	96.1	96.0
Type of neonatal complication				
**Asphyxia**	5.4	5.7	2.4	2.1
**Still birth**	2.7	2.9	0.9	0.8
**Infection**	1.5	1.0	0.4	0.8
**Early neonatal death**	1.0	0.6	0.4	0.2

Other maternal complications*: prolonged second stage of labor, tear and vomiting

### Characteristics of skilled birth attendants

At baseline, 121 skilled birth attendants or providers completed the knowledge and clinical vignettes. By end line, of those providers who participated in the baseline survey, 112 (92.56%) completed the knowledge test and the vignettes. About six out of ten health care providers at baseline control group and end line intervention group had less than five years work experience. Almost sixty percent of the providers had diploma degree in both control groups of the survey. Of SBAs working in their local intervention facility at baseline, 51.4% reported they were satisfied with their job, compared to 65% of providers in intervention facilities at end line. Among providers who completed the knowledge test at baseline control group, 62.7% had in adequate knowledge and 57.7% in the intervention group follow-up had adequate knowledge on maternal and newborn health [Table 3].

**Table 3:** Characteristics of skilled birth attendants working in primary health facilities of Tigray, Northern Ethiopia, 2020 (N = 236).

Demographics	Baseline		End line	
	Control Mean or percent	Intervention Mean or percent	Control Mean or percent	Intervention Mean or percent
Age (mean)	29.24	29.67	28.5	27.85
Work experience				
Less than five years	60.8	45.7	60.0	61.5
Five years and above	39.2	54.3	40.0	38.5
Sex				
Female	64.7	61.4	63.3	73.1
Male	35.3	38.6	36.7	26.9
Highest level of education				
Diploma	60.8	48.6	61.7	40.4
Degree and above	39.2	51.4	38.3	59.6
Educational program attended				
Generic	68.6	68.6	78.3	65.4
Upgrade regular	21.6	15.7	10.0	19.2
Upgrade in-service	9.8	15.7	11.7	15.4
Marital status				
Single	39.2	21.4	16.7	38.5
Married	45.1	72.9	476.7	57.7
Divorced	15.7	5.7	6.7	3.8
Cadre				
Midwife	60.8	50.0	81.7	84.6
Nurse	29.4	28.6	15.0	9.6
Health officer	9.8	21.4	3.3	5.8
Have regular case presentation in the facility or case team				
Yes	51.0	41.4	65.0	71.2
Have fear of legal issue to make decision in daily basis				
Yes	15.7	25.7	30.0	21.2
Have challenge in providing intra-partum, and immediate postpartum care				
Yes	37.3	32.9	45.0	30.8
Postnatal women checked and discharged by senior staff of the facility				
Yes	29.4	21.4	31.7	46.2
Providers received trainings in the last 2 years (Yes):				
Basic Emergency Obstetrics and Newborn care	64.7	62.9	35.0	42.3
Neonatal resuscitation	52.9	42.9	33.3	48.1
Compassionate and respectful maternity care	37.3	31.4	41.7	38.5
Quality improvement initiatives	13.7	22.9	21.7	36.
Delivery load at weekly basis (mean)	3.9	4.3	10.7	13.7
Providers of satisfaction				
Satisfied	49.0	51.4	53.3	65.4
Providers knowledge				
Adequate knowledge	37.3	40.0	46.7	57.7
Clinical vignette				
Competent	52.9	50.0	53.3	63.5
Motivation				
Motivated	21.6	28.6	23.3	42.3

### Facility characteristics

At both survey intervention groups, more than 80% of the facilities had implemented maternal, perinatal/neonatal death surveillance and responding (MPNDSR). For the control group at baseline and end line, more than four fifth and three fifth of the health facilities had not promoted regular staff rotation respectively. More than three quarters of the facilities during baseline assessment across the groups were inadequately ready but 77.8% of the end line intervention facilities were adequately ready [Table 4].

**Table 4:** Characteristics of study facilities in Northern Ethiopia, 2020 (n = 19).

Variables	Baseline		End line	
	Control Mean or percent	Intervention Mean or percent	Control Mean or percent	Intervention Mean or percent
Maternal, perinatal/neonatal death surveillance and responding (MPNDSR)				
Yes	77.8	80.0	77.8	88.9
No	22.2	20.0	22.2	11.1
Promotes staff rotation				
Yes	15.7	21.4	40.0	44.2
No	84.3	78.6	60.0	55.8
Collected data of maternal and newborn health regularly				
Yes	55.6	40.0	77.8	100.0
No	44.4	60.0	22.2	0.0
Mobile data internet access				
Yes	66.7	60.0	88.9	88.9
No	33.3	40.0	11.1	11.1
Maternal Newborn Health quality improvement initiative				
Yes	22.2	30.0	33.3	66.7
No	77.8	70.0	66.7	33.3
Facility readiness				
Adequately ready	22.2	20.0	55.6	77.8
Inadequately ready	77.8	80.0	44.4	22.2

### Effect of the intervention

**Fig 3:**
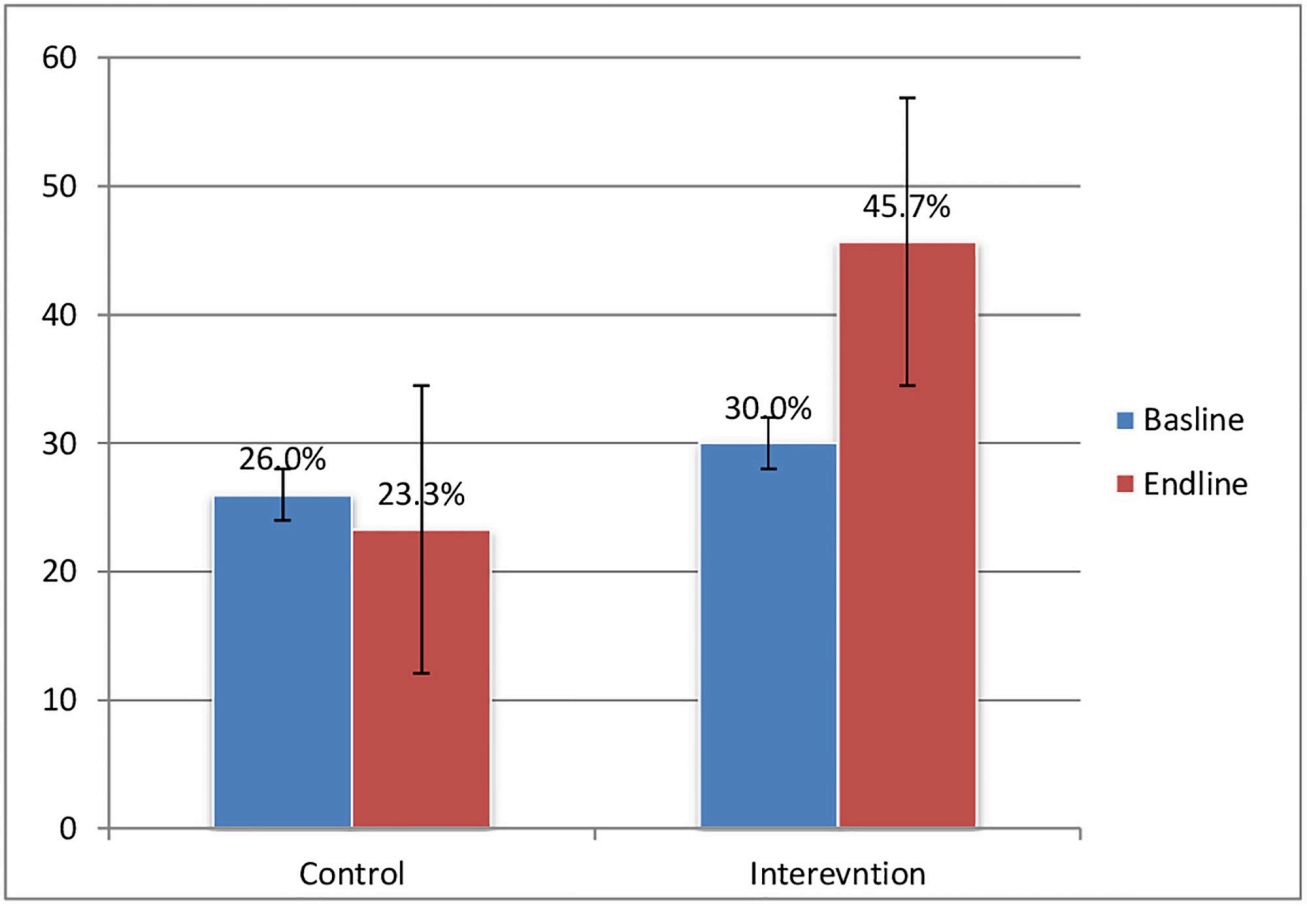
Proportion of mothers and newborns received quality of care during intrapartum and immediate postpartum period, baseline and post intervention, 2019 and 2020.

Difference in differences analysis the effect of the catchment based mentorship approach intervention shown in “Fig 3” revealed that the proportion of mothers who received QoC at end line in the control group was 30.0% compared to 45.7% in the intervention group. The DiD analysis found significant differences in QoC in the intervention facilities, which is 18% increase, compared to the control facilities (DiD = 18.4%, p< 0.001).

A significant higher proportion of women at intervention facilities received quality of care services, compared with women at comparison primary health care facilities. After the intervention was implemented the occurrences of maternal complications following delivery and immediately after birth was decreased by 4.5%, with significant differences between intervention and comparison sites (DiD = 4.5%, p = 0.013). We also found a favorable DiD among neonatal outcomes, in terms of, occurrences of negative neonatal obstetric complications, with a decrease of 4.8% at the intervention sites and almost no change in the comparison sites (DiD = 4.8%, p = 0.002) [Table 5].

**Table 5:** DID analysis of the effect of intervention between intervention and control facilities at baseline and end line, northern Ethiopia.

Indicators	Intervention arm			Control arm				
	Baseline (n, %)	End line (n, %)	Diff.^1^(Ie-Ib)	Baseline (n, %)	End line (n, %)	Diff-^2^(Ce-Cb)	DiD (Diff ^1^- Diff^2^)	p- value
Mother’s received quality of care	77(23.3)	150(45.7)	22.4	86(26.0)	99(30.0)	4.0	18.4	0.001
Maternal and newborn complications following condition either during or immediately following delivery								
Maternal obstetric complication (yes to any)	37(11.2)	19(5.8)	5.4	33(10.0)	30(9.1)	0.9	4.5	0.013
Neonatal complication(yes to any)	34(10.3)	13(4.8)	5.5	35(10.6)	32(9.9)	0.7	4.8	0.002

Ie: intervention end line; Ib: intervention baseline; Diff.

^1^ difference; Ce: control end line; Cb: control baseline; DiD, difference-in-difference.

Maternal obstetric complication; hemorrhage, Pregnancy induced hypertension, infection, tear and maternal death at facility.

Neonatal complication; birth asphyxia, still birth, infection and newborn death.

### Predictors of quality of maternal and newborn care in the catchment based clinical mentorship intervention

Variables, such as group status (mentorship intervention), job satisfaction, making regular case presentation, and delivery load were significant determinants of QoC.

The odds of provision of QoC were two times higher among those skilled birth attendants (SBAs) who received the intervention when compared to those comparison group of providers (AoR = 1.86, 95%CI: 1.40, 2.85).

Similarly, satisfied SBAs were three times higher to provide QoC for mothers and newborns during intra-partum and immediate postpartum than their dissatisfied counterparts (AoR = 2.95, 95%CI: 1.26, 6.91). The likelihood of provision of QoC among those providers who had case presentations on a regular basis was two times higher than those who did not have case presentations on a regular basis (AoR = 1.89, 95%CI: 1.05, 3.39).

Furthermore, as the delivery load of the providers increases by one unit, the QoC provision decreases by 5% (AoR = 0.95, 95%CI: 0.93, 0.98) [Table 6].

**Table 6:** Multivariable logistic regression with GEE analysis results of predictors of quality of care in Northern Ethiopia, 2020.

Variable	Quality of care (n, %)				Odds Ratio, 95%CI	
	Baseline		End line			
	In-adequate	Adequate	In-adequate	Adequate	Crude	Adjusted
Group status of participants						
Intervention	254(76.7)	77(23.3)	178(54.3)	150(45.7)	1.50(1.14,1.96)	**1.86(1.40,2.85)** *
Control	245(74.0)	86(26.0)	231(70.0)	99(30.0)	1	
Health providers work experience (in years)						
Five and above	43(74.1)	15(25.9)	22(50.0)	22(50.0)	0.88(0.45,1.71)	
Less than five	31(49.2)	32(50.8)	27(39.7)	41(60.3)	1	
Providers’ sex						
Female	47(61.8)	29(38.2)	32(42.1)	44(57.9)	0.76(0.38,1.54)	
Male	27(60.0)	18(40.0)	17(47.2)	19(52.8)	1	
Providers education						
Degree and above	35(62.5)	21(37.5)	20(37.0)	34(63.0)	0.76(0.39,1.48)	
Diploma	39(60.0)	26(40.0)	29(50.0)	29(50.0)	1	
Education program attended						
Generic	49(59.0)	34(41.0)	36(44.4)	45(55.6)	1.27(0.48,3.39)	
Upgrade regular	15(68.2)	7(31.8)	6(37.5)	10(62.5)	1.38(0.41,4.62)	
Upgrade in-service	10(62.5)	6(37.5)	7(46.7)	8(53.3)	1	
Cadre						
Midwife	46(69.7)	20(30.3)	41(44.1)	52(55.9)	3.26(0.65,16.39)	0.17(0.02,1.43)
Nurse	17(48.6)	18(51.4)	6(42.9)	8(57.1)	2.23(0.36,13.96)	0.98(0.29,3.36)
Health officer	11(55.0)	9(45.0)	2(40.0)	3(60.0)	1	
Have case presentation at regular basis						
Yes	32(58.2)	23(41.8)	13(36.1)	40(52.6)	1.56(0.78,3.14)	**1.89(1.05,3.39)** *
No	42(63.6)	24(36.4)	13(36.1)	23(63.9)	1	
Fear of legal issues to make decisions						
Yes	18(69.2)	8(30.8)	9(31.0)	20(69.0)	1.88(0.87,4.07)	2.27(0.98,5.26)
No	56(58.9)	39(41.1)	40(48.2)	43(51.8)	1	
Received maternal and newborn health (MNH) related trainings^1^						
Yes	66(60.6)	43(39.4)	32(42.7)	43(57.3)	0.63(0.29,1.31)	0.58(0.55,1.26)
No	8(66.7)	4(33.3)	17(45.9)	20(54.1)	1	
Satisfaction of providers						
Satisfied	39(63.9)	22(36.1)	32(49.2)	33(50.8)	2.55(1.24,5.25)	**2.95(1.26,6.91)** *
Dissatisfied	35(58.3)	25(41.7)	17(36.2)	47(38.8)	1	
Providers knowledge						
Adequate	26(55.3)	21(44.7)	25(43.1)	33(56.9)	2.03(1.04,3.99)	1.9(0.95,3.79)
Inadequate	48(64.9)	26(35.1)	24(44.4)	30(55.6)	1	
Vignette						
Competent	41(66.1)	21(33.9)	30(46.2)	35(53.8)	1.60(0.82,3.14)	1.47(0.68,3.18)
Not yet competent	33(55.9)	26(44.1)	19(40.4)	28(59.6)	1	
Motivation						
Motivated	20(64.5)	11(35.5)	15(41.7)	21(58.3)	1.02(0.50,2.06)	
De-motivated	54(60.0)	36(40.0)	34(44.7)	42(55.3)	1	
Promote staff rotation						
Yes	18(78.3)	5(21.7)	17(36.2)	30(63.8)	1.03(0.42,2.48)	1.72(0.87,3.38)
No	56(57.1)	42(42.9)	32(49.2)	33(50.8)	1	
Delivery load	4.2±3.19	3.93±2.37	23.87±11.02	20.2±8.74	0.98(0.95,1.00)	**0.95(0.93,0.98)** *
Facility has maternal, perinatal, neonatal death surveillance and responding (MPNDSR)						
Yes	6(40.0)	9(60.0)	1(12.5)	7(87.5)	1.06(1.0,1.08)	
No	2(50.0)	2(50.0)	2(20.0)	8(80.0)	1	
Facility has mobile data internet access						
Yes	5(41.7)	7(58.3)	3(18.8)	13(81.2)	1.13(0.06,21.09)	
No	3(42.9)	4(57.1)	0(0.0)	2(100)	1	
Facility has maternal, newborn health quality improvement initiative						
Yes	4(80.0)	1(20.0)	2(22.2)	7(77.8)	0.53(0.09,3.31)	
No	4(28.6)	10(71.4)	1(11.1)	8(88.9)	1	
Facility readiness						
Adequately ready	3(75.0)	1(25.0)	2(16.7)	10(83.3)	0.75(0.12,4.91)	
Inadequately ready	5(33.3)	10(66.7)	1(16.7)	5(83.3)	1	

^**1**^Four types of training(BEmONC, NR, RMC and QI),

* shows statistical significance at p-value less than 0.05.

## Discussion

We conducted a quasi-experimental study to assess the effectiveness of a catchment based clinical mentorship program to improve quality of maternal and newborn health care in the PHC level of northern Ethiopia, Tigray regional state. A catchment based clinical mentorship intervention is a system of practical training and consultation that enables health care provides to practice new skills in clinical settings with the support and guidance of a more specialized and experienced clinician to yield sustainable, high-quality clinical care outcomes [16]. In catchment-based mentorship, the mentors were selected from within the existing health care system and are being responsible to mentoring the facilities within their catchment.

Our study showed that following the catchment based mentoring program, a differences in the quality of services provided. This indicated that, women delivering at intervention primary health facilities in Tigray, northern Ethiopia received significantly improved QoC, compared with women who delivered at comparison facilities. This finding indicated an 18% increase in QoC at the intervention facilities, compared to the control facilities. Following the implementation of the intervention the occurrences of maternal complication outcome during delivery and immediately after birth was decreased by 4.5% in the intervention sites. We also found a favorable difference in occurrences of negative neonatal obstetric complications, with a decrease of 4.8% in the intervention sites, and almost no change in the comparison sites. Among the determinants of QoC, we found that catchment based mentorship intervention, job satisfaction, and making case presentation at regular basis were significantly associated to improve the quality of maternity care services. However, delivery load is negatively associated with QoC. These findings reflect the need for better job satisfaction;scale-up of catchment based mentorship program, more attention and thoughtful schemes are required in deploying the number of skilled birth attendants in relation to the delivery load per facilities. Other studies had similar conclusions [7, 28, 33].

Our study revealed that, the provision of QoC was higher among those SBAs received catchment based mentorship when compared to those non-mentored providers. This finding indicates that facilities who had mentorships performed much better than the health centers that did not go through a mentoring program. Hence, CBCM is an effective intervention in improving QoC process at time of childbirth, which, in turn, improves MNH outcomes. This result is supported by studies done in Rwanda [17] and India [29, 34].

Similarly, there were significant reductions in the proportion of maternal and newborn complications at the intervention facility with no change at the control facility. This study result is consistent with studies done in Rwanda [14, 17]. This implies that catchment based clinical mentoring intervention may be an important component in adhering to obstetrics standard guidelines, improving clinical competencies and caring behaviors and skills to provide the routine MNH care functions which in turn improve the overall QoC services.

We also found that, the likelihood of provision of quality of care among those providers who experienced job satisfaction was higher than those who were unsatisfied. This finding is comparable with the studies done in Pakistan [35]. Hence; more attention is needed to develop interventions and strategies that directly enhance provider satisfaction in various contexts to improve QoC. Similarly, in this study, the provision of QoC has a direct relationship with providers’ making case presentation on a regular basis. This finding is congruent with the studies done in Ethiopia [7], which indicates that the SBAs were continually earning a new knowledge during case presentation. However, delivery load is linked with significant decreases in QoC. This finding was complemented by the study done in Ethiopia [7] that revealed high delivery load were major bottlenecks in the provision of timely and quality obstetric and new born care, which has a significant impact on maternal and neonatal outcomes. Therefore, efforts and attentive strategies that match providers to workload are necessary to mitigate the effects of working in this context and to improve the quality of obstetric care.

We distinguish a number of limitations of our study. Firstly, the study implemented a quasi-experimental trial design, introducing the potential for selection bias. However, we applied matching principle—a statistical procedure—to match the intervention and comparison facilities on a number of variables, so that the facilities were as comparable as possible. Secondly, difficulty maintaining the cohort of mentees due to frequent staff transfers and maternity or pregnancy leaves were noted. Thirdly, it lacks the finding report of process evaluation. Fourthly, near the completion of the intervention, the efficacy and full implementation of the intervention might have been affected by the pandemic COVID -19 and lockdown measures. Lastly, although this study was conducted before the dreadful war happened in Tigray, those results might not be easy applicable for the current health system of Tigray. As currently the ongoing armed conflict has caused devastating and collapsed health system, in which more than 70% of health facilities were damaged, vandalized and looted by the allied forces. Therefore, readers needs a great attention while interpret these findings.

Nonetheless, this interventional study is one of the first to assess the effect of CBCM to improve QoC services for mothers and newborns in Ethiopia. The project was able to secure the involvement of the Regional Health Bureau/implementers with the research team members from its planning to the evaluation phase

## Conclusions

This study concluded that the catchment based clinical mentorship program is effective to improve the QoC and reduction of childbirth complications. This finding further elaborated that incorporating MNH CBCM activities into the existing health system strengthening strategies can catalyze improvement processes to quality practice and health systems in resource poor settings. This is seen as a necessary step to achieve the effective quality universal health care required to meet the health-related Sustainable Development Goals. Furthermore, we found that providers’ job satisfaction, making case presentation on a regular basis and delivery load were contributory to QoC. Efforts must be undertaken to sustain QoC through institutionalizing strict CBCM across all PHC maternity settings to comply with the standardized protocol of clinical practice. Since catchment based SBAs mentoring program have emerged as an attractive intervention for training front line midwives, obstetric nurses and health officers in rural PHCs, the lessons from this study may help in addressing some of the challenges of QoC. Women may bypass poor quality PHCs and overburden higher level facilities that may adversely affect treatment of women in need of emergency obstetric care at referral hospitals. Therefore, more attention needs to be given to develop interventions and strategies that directly enhance provider satisfaction and reduce delivery work load to improve QoC.

Finally, although we draw a conclusion about the significant effects of mentorship, another study was also required to determine how we sustain the program, for how long the mentorship should be sustained, and or how long will this effect last once the mentorship is stopped.

## Supporting information

S1 Appendix Recruitment and selection criteria for local SBA mentors in CBCM intervention in Northern Ethiopia. (DOCX)

S2 Appendix Results of the chi-squared test of homogeneity between intervention and comparison groups among ABAs from the baseline survey. (DOCX)

S1 Dataset Supporting information, data set on the effect of intervention. (SAV)

## Abbreviations

AoR: adjusted odds ratio
DiD: difference–in-difference
GEE: generalized estimating equation
MNH: maternal and newborn health
QIC: quasilikelihood under the independence model information criterion
QoC: quality of care
SBA: skilled birth attendants
