# Supplementary material for: The effect of catchment based mentorship on quality of maternal and newborn care in primary health care facilities in Tigray Region, Northern Ethiopia: A controlled quasi-experimental study
Source: PLoS One. 2022 Nov 17;17(11):e0277207. doi: 10.1371/journal.pone.0277207 (PMC9671353; doi:10.1371/journal.pone.0277207)
Supplement: S1 Appendix — (DOCX) [file pone.0277207.s001.docx]

**S1 Appendix: Recruitment and Selection criteria for local SBA mentors for CBCM intervention in Northern Ethiopia**

| **Number** | **Indicators** |
| --- | --- |
| 1 | Educational qualification with relevant clinical work experience (at least bachelor’s degree/BSc with a minimum of three years’ experience) |
| 2 | Demonstrated willingness to mentor other clinicians in their district or catchment area through on-site visits |
| 3 | Capacity and desire to motivate the mentee to perform well measured using performance evaluation of the previous months |
| 4 | Qualified, competent and experienced in own area of specialization with clinical proficiency and capacity to make decisions |
| 5 | Familiarity with and ability to conduct procedures in accordance with clinical standards and guidelines or received relevant trainings like BEmONC, Essential newborn care etc… |
| 6 | Ability to facilitate a case discussion including testimony of clinical-teaching work experience. |
| 7 | Aptitude result of the individual counted by a cumulative result of the given written test and interview. |
| 8 | Ability to communicate clearly and effectively with staff including provision of constructive, timely, and interactive feedback |
| 9 | Ability to gather, analyze data and report accordingly |
| 10 | Be a role model and champion of best practices within their own facility |
| 11 | Being available and committed to mentorship |
| 12 | Demonstrated ability to transfer knowledge and skills |
| 13 | Interested in clinical mentorship |
