# Supplementary material for: The effect of catchment based mentorship on quality of maternal and newborn care in primary health care facilities in Tigray Region, Northern Ethiopia: A controlled quasi-experimental study
Source: PLoS One. 2022 Nov 17;17(11):e0277207. doi: 10.1371/journal.pone.0277207 (PMC9671353; doi:10.1371/journal.pone.0277207)
Supplement: S2 Appendix — (DOCX) [file pone.0277207.s002.docx]

**S2 Appendix results of the chi-squared test of homogeneity between intervention and comparison groups among SBAs from the baseline survey**

| Demographics | | Baseline survey | | |
| --- | --- | --- | --- | --- |
|  |  | **Control**  **Mean/ percent** | **Intervention**  **Mean/ percent** | **p-value** |
| Age (mean) | 29.24 | | 29.67 | 0.56 |
| Work experience | | | | |
| Less than five years | 60.8 | | 45.7 | 0.10 |
| Five years and above | 39.2 | | 54.3 |  |
| Sex | | | | |
| Female | | 64.7 | 61.4 | 0.71 |
| Male | | 35.3 | 38.6 |  |
| Highest level of education | | | | |
| Diploma | | 60.8 | 48.6 | 0.15 |
| Degree and above | | 39.2 | 51.4 |  |
| Educational program attended | | | | |
| Generic | | 68.6 | 68.6 | 0.51 |
| Upgrade regular | | 21.6 | 15.7 |  |
| Upgrade in-service | | 9.8 | 15.7 |  |
| Marital status | | | | |
| Single | | 39.2 | 21.4 | 0.01 |
| Married | | 45.1 | 72.9 |  |
| Divorced | | 15.7 | 5.7 |  |
| Cadre | | | | |
| Midwife | | 60.8 | 50.0 | 0.22 |
| Nurse | | 29.4 | 28.6 |  |
| Health officer | | 9.8 | 21.4 |  |
| Have regular case presentation in the facility or case team | | | | |
| Yes | | 51.0 | 41.4 | 0.29 |
| No | | 49.0 | 58.6 |  |
| Have fear of legal issue to make decision in daily basis | | | | |
| Yes | | 15.7 | 25.7 | 0.18 |
| No | | 84.3 | 74.3 |  |
| Have challenge in providing intra-partum, and immediate postpartum care | | | | |
| Yes | | 37.3 | 32.9 | 0.62 |
| No | | 62.7 | 67.1 |  |
| Postnatal women checked and discharged by senior staff of the facility | | | | |
| Yes | | 29.4 | 21.4 | 0.32 |
| No | | 70.6 | 78.6 |  |
| Providers received trainings in the last 2 years (Yes): | | | | |
| Basic Emergency Obstetrics and Newborn care | | 64.7 | 62.9 | 0.84 |
| Neonatal resuscitation | | 52.9 | 42.9 | 0.27 |
| Compassionate and respectful maternity care | | 37.3 | 31.4 | 0.64 |
| Quality improvement initiatives | | 13.7 | 22.9 | 0.21 |
| Delivery load at weekly basis (mean) | | 3.9 | 4.3 | 0.32 |
| Providers’ of satisfaction | | | | |
| Satisfied | | 49.0 | 51.4 | 0.79 |
| Not satisfied | | 51.0 | 48.6 |  |
| Providers knowledge | | | | |
| Adequate knowledge | | 37.3 | 40.0 | 0.76 |
| Inadequate knowledge | | 62.7 | 60.0 |  |
| Clinical vignette | | | | |
| Competent | | 52.9 | 50.0 | 0.75 |
| Not yet competent | | 47.1 | 50.0 |  |
| Motivation | | | | |
| Motivated | | 21.6 | 28.6 | 0.38 |
| De-motivated | | 78.4 | 71.4 |  |
